# Greater numbers and sizes of muscle bundles in the breast and leg muscles of broilers compared to layer chickens

**DOI:** 10.3389/fphys.2023.1285938

**Published:** 2023-10-09

**Authors:** Boin Lee, Dong-Hwan Kim, Joonbum Lee, Michael D. Cressman, Young Min Choi, Kichoon Lee

**Affiliations:** ^1^ Department of Animal Sciences, The Ohio State University, Columbus, OH, United States; ^2^ Department of Animal Science and Biotechnology, Kyungpook National University, Sangju-si, Gyeongsangbuk-do, Republic of Korea

**Keywords:** broiler, layer, muscle hyperplasia, muscle hypertrophy, muscle bundle

## Abstract

Meat-type (broiler) and egg-type (layer) chickens were bred by intensive selection over the years, resulting in more numbers and larger sizes of myofibers. Although the characteristics are important parameters in muscle growth and meat quality, muscle bundle characteristics have not been studied in poultry. Therefore, this study aimed to compare the histological characteristics of myofibers and muscle bundles in muscles between male broiler (Ross broiler breed) chickens and layer (Hy-Line) chickens. Chicken muscles, *pectoralis major* (PM) and *gastrocnemius* (GM), were sampled at the age of 49 days and stained to analyze histological characteristics. Expectedly, body weights (BWs) and weights of PM and GM muscles in 49-day-old broilers were significantly heavier than those in layers. Within PM, broilers exhibited greater number and cross-sectional area (CSA) of myofibers than layers (3.3- and 3.3-fold, respectively). The total number and CSA of PM muscle bundles were approximately 1.5 and 6.6 times greater, respectively, in broilers than layers. Moreover, broilers exhibited 2 times greater number of myofibers per bundle of PM muscle than layers. Within GM, myofiber number and CSA were 2.3- and 2.4-fold greater, respectively, in broilers than layers. In addition, the total number of muscle bundles and bundle CSA were 2.5- and 2.1-fold greater, respectively, in broilers than in the layers. The novel findings of the current study provide evidence that greater muscle mass of broilers occurs by both hyperplasia and hypertrophy of muscle bundles and myofibers.

## 1 Introduction

Poultry meat and egg consumption are steadily increasing as consumers face increasing health concerns related to red meat consumption. As a meat-type poultry breed, broilers have been genetically selected to have a fast-growing performance and high meat yield, whereas layers selected for egg production and backyard chickens, such as Hubbard JA57, have a slow-growing performance. As a result, they show significant differences in body and muscle weight, especially in the breast muscle, and these differences most likely involve the quantity or size of myofibers (Scheuermann et al., 2004).

Muscle bundle characteristics have been compared and related with growth characteristics of muscle among different breeds of livestock species (Albrecht et al., 2006). Greater muscle mass in fast-growing animals is generally associated with increased number (hyperplasia), increased size (hypertrophy), or both of myofibers and bundles, which can be attributed to various factors, such as animals, species, body weight, breed, age, sex, growth rate, and physical activity (Kiessling, 1977; Scheuermann et al., 2004; Albrecht et al., 2006; Choi and Kim, 2009; Choi et al., 2013a; Choi et al., 2013b; Choi et al., 2014; Kokoszyński et al., 2018; Kokoszyński et al., 2022; Kim et al., 2022; Weng et al., 2022). The low-weight quail line exhibited a lower number, but similar size of myofibers, compared to the random bred control (RBC) quail line, providing a unique muscle hypoplasia model in avian species (Choi et al., 2014). In contrast, the heavy-weight quail line has a greater size of myofibers in the breast muscle, but no difference in total fiber number compared to the RBC quail line (Choi et al., 2013b). Additionally, myofiber hypertrophy appeared in the fast-growing duck line compared to ducks in the slow-growing line (Huo et al., 2021). In chickens, commercial broiler lines with higher breast yield compared to Leghorn egg-type chickens of the same age and sex showed myofiber hyperplasia and hypertrophy (Scheuermann et al., 2004). Although muscle bundle characteristics are important parameters contributing to muscle growth and meat quality in livestock animals (Scheuermann et al., 2004; Albrecht et al., 2006; Chandraratne et al., 2006; Lee et al., 2018; Choi et al., 2019), these factors have not been extensively studied in chickens. Therefore, the objective of this study was to compare histological traits of myofiber and muscle bundle in the pectoralis major (PM) and gastrocnemius (GM) muscles between male broiler chickens and layer chickens.

## 2 Materials and methods

### 2.1 Animal care

Commercially available chickens (broiler and layer; Ross broiler breed and Hy-Line, respectively) and experiments were approved by The Ohio State University Institutional Animal Care and Use Committee (IACUC; protocol no. 2020A00000094). All animals were raised under the same environmental conditions such as room temperature and the size of brooder cages. In addition, we fed the same diet to both layer and broiler chickens to eliminate diet effects (Table 1). Chickens were euthanized by cervical dislocation after CO_2_ inhalation according to the IACUC protocol.

**TABLE 1:** Ingredient and calculated nutrient composition of the dry matter of diets fed to both layer and broiler chickens.

Corn (kg 100 kg^-1^)	41.9
Soybean meal, 48% (kg 100 kg^-1^)	44.4
Meat and bone meal, 55% (pork) (kg 100 kg^-1^)	5
Blended fat (kg 100 kg^-1^)	2.9
D,L-Methionine (kg 100 kg^-1^)	0.25
L-Lysine (kg 100 kg^-1^)	0.15
Salt (kg 100 kg^-1^)	0.4
Limestone (kg 100 kg^-1^)	0.7
Dicalcium phosphate, 18.5% (kg 100 kg^-1^)	2.85
Copper sulfate, fine 25.2% (kg 100 kg^-1^)	0.05
Amprolium, 2.5% (kg 100 kg^-1^)	1
Selenium, 90.8 mg/lb (kg 100 kg^-1^)	1
Choline chloride (kg 100 kg^-1^)	0.15
L-Lysine (kg 100 kg^-1^)	0.15
Vitamin A (IU/kg^-1^)	13,200

### 2.2 Collection of muscle samples

A total of 12 male chickens (age, 49 days; broiler, n = 6; layer, n = 6) were used in this study. Body weight (BW), PM muscle weight (PMW), and GM muscle weight (GMW) were measured, and percentages of PMW and GMW were calculated in relation to BW. After measurement of PMW, CSA of the left PM muscle was measured in an area cut from the lower left to the upper right at the half-point of the muscle (Scheuermann et al., 2004). Whole right GM muscles were fixed and then cut in the middle of the muscle to prepare paraffin blocks.

### 2.3 Histological processing and measurement of myofibers and muscle bundles

PM and GM muscles fixed with 10% neutral-buffered formalin were embedded in paraffin and then cross-sectioned into 10-µm slices. The sections were stained using a hematoxylin and eosin stain method following our previous study (Kim et al., 2022). All stained sections were assessed in terms of myofiber and muscle bundle characteristics, including total number, average CSA, and myofiber number per bundle, using image analysis (Image-Pro Plus software, Media Cybernetics, Silver Spring, MD). For each sample, at least 500 different fibers and 30 bundles were randomly selected and measured to determine these parameters at ×10 and ×40 magnification. Average CSA and total number of myofibers were calculated according to previous studies (Scheuermann et al., 2004; Kim et al., 2022). Total bundle number was calculated by dividing the PM muscle CSA by the mean bundle area of each sample. The average of the bundle CSA was determined by dividing the total bundle area by the total bundle number measured.

### 2.4 Statistical analysis

To compare carcass traits and histological characteristics between broiler and layer chickens, the data were analyzed by *t*-tests using GraphPad Prism software, version 6.02. All data were expressed as means ± SEM. The results with *p* < 0.05 were considered significant.

## 3 Results

As expected, at 49 days post-hatch, broilers exhibited heavier body weight than layers (4,097 vs 512.3 g, *p* < 0.001) (Table 2). PMW (946.7 vs 36.5 g, *p* < 0.001) and percentage of PM (23.1% vs 7.06%, *p* < 0.001) were 25.9- and 3.3-fold greater in broilers than in layers, respectively. GMW of the broiler was approximately 8.3 times greater than that of the layer (41.3 g vs 4.98 g, *p* < 0.001), although there was no difference in the percentage of the GM muscle between the breeds (1.01% vs 0.97%, *p* > 0.05).

**TABLE 2:** Comparison of body weight, carcass traits, and histological traits of the *pectoralis major* and *gastrocnemius* muscles between broiler and layer chickens at 49 days post-hatch.

	Broiler (n = 6)	Layer (n = 6)	Level of significance
*Weight and carcass traits*			
Body weight (g)	4,097 (396.3)^1^	512.3 (67.7)	***
PM muscle weight (g)	946.7 (112.9)	36.5 (8.63)	***
PM muscle percentage (%)	23.1 (1.19)	7.06 (0.64)	***
GM muscle weight (g)	41.3 (5.45)	4.98 (0.62)	***
GM muscle percentage (%)	1.01 (0.09)	0.97 (0.06)	NS
*Histological traits of PM muscle*			
Total myofiber number (× 1,000)	1,380 (442.1)	415.2 (141.2)	**
Myofiber CSA (μm^2^)	3,377 (1 330)	1,026 (170.5)	**
Total bundle number	4,941 (1,175)	3,215 (1,174)	*
Bundle CSA (μm^2^, × 1,000)	906.0 (291.1)	138.1 (38.9)	***
Myofiber number per bundle	276.7 (47.6)	138.5 (46.4)	***
*Histological traits of GM muscle*			
Total myofiber number (× 1,000)	308.2 (82.8)	134.5 (24.1)	***
Myofiber CSA (μm^2^)	1,400 (368.2)	589.2 (95.0)	***
Total bundle number	2,937 (670.1)	1,152 (52.4)	***
Bundle CSA (μm^2^, × 1,000)	143.7 (29.0)	67.6 (7.00)	***
Myofiber number per bundle	106.1 (22.1)	116.5 (17.3)	NS

Level of significance: NS, no significance; * *p* < 0.05; ** *p* < 0.01; *** *p* < 0.001.

^a^ Standard error of least-square means.

PM, *pectoralis major*; GM, *gastrocnemius*; CSA, cross-sectional area.

**FIGURE 1:**
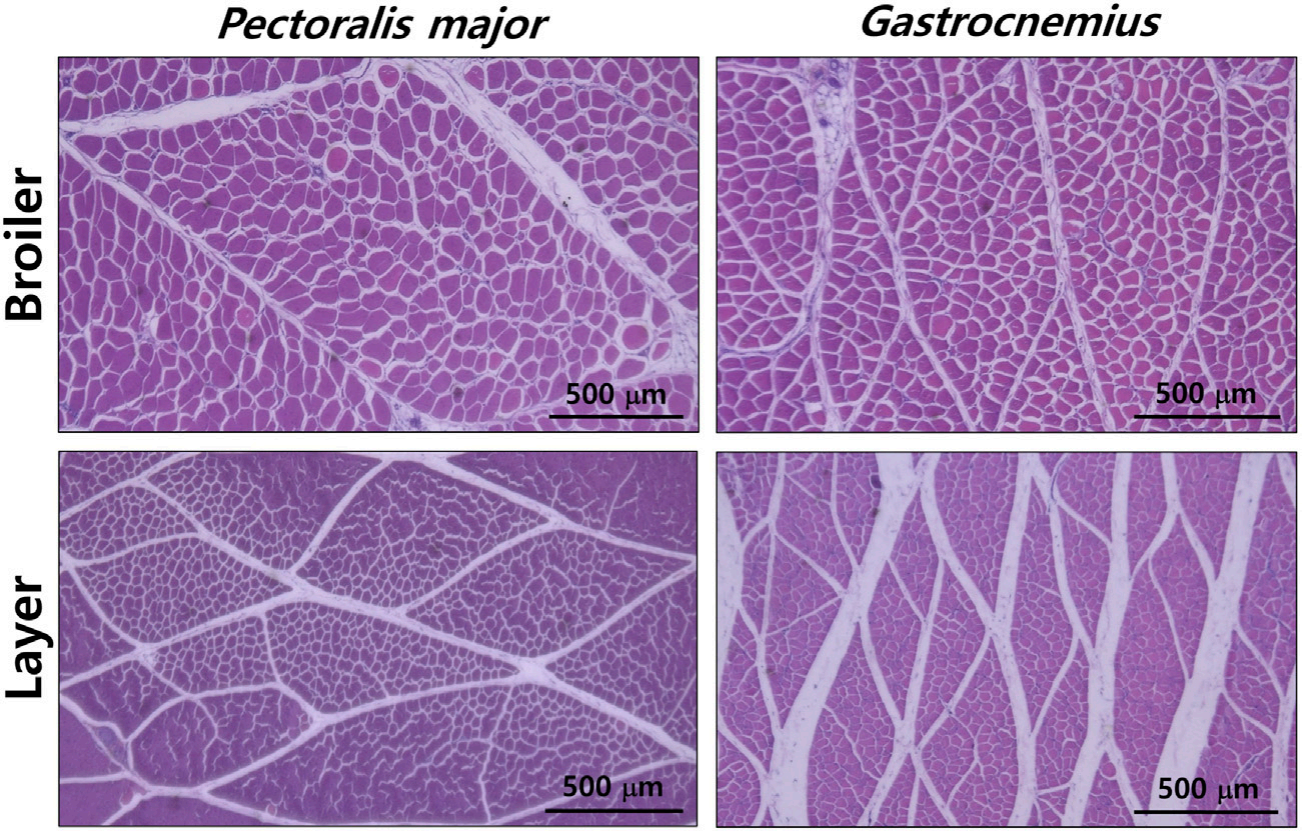
Histological differences in *pectoralis major* and *gastrocnemius* muscles between male broiler and layer chickens at 49 days post-hatch. Scale bar: 500 μm.

PM muscles of broiler chickens had a 3.3-fold greater total number (1,380,000 vs 415,200, *p* < 0.01) and CSA (3,377 vs 1,026 μm^2^, *p* < 0.01) of myofibers compared to those of layer chickens. In the GM muscle, broilers showed more number (308,200 vs 134,500, *p* < 0.001) and greater size (1,400 vs 589.2 μm^2^, *p* < 0.001) of myofibers compared to layers. PM muscles of broilers were 1.5-, 6.5-, and 2.0-fold greater in total bundle number (4,941 vs 3,215, *p* < 0.05), bundle CSA (906,000 vs 138,100 μm^*2*^, *p* < 0.001), and myofiber number per bundle (276.7 vs 138.5, *p* < 0.001), respectively, compared to those of layers. Similar to PM muscles, greater number (2,937 vs 1,152, *p* < 0.001) and CSA (143,700 vs 67,600 μm^2^, *p* < 0.001) of GM muscle bundles were found in broilers compared to layers; whereas two breeds did not differ in myofiber number per bundle (106.1 vs 116.5, *p* > 0.05). Their representative images are presented in Figure 1.

## 4 Discussion

It is generally reported that fast-growing broiler chickens are 2- to 3-fold greater in growth rate, and in particular, their breast muscles grow 8-fold faster than those in layer-type chickens (Buzala and Janicki, 2016). The findings that greater percentage of PM muscle in broilers, but similar percentage of GM muscle between two breeds, clearly provide evidence for the general selection for greater yield of breast muscle but not for leg muscle. Our previous study reported that 33-day-old broiler chickens had 2.7-fold greater myofiber CSA of PM muscle than layer chickens at the same age (Kim et al., 2022). In addition, myostatin knock-out chickens showing a rapid growth rate exhibited heavier body weight and greater myofiber CSA of the *semitendinosus* muscle than wild-type chickens with a slower growth rate (Kim et al., 2020). Similar to these results, in this study, broilers showing a heavier body weight had a greater total number and CSA of myofibers of the PM and GM muscles compared to layers showing a lighter body weight. This suggests that greater PMW and GMW of broilers could have resulted from both myofiber hyperplasia and hypertrophy.

A muscle fascicle is a bundle of different numbers of myofibers surrounded by connective tissue (Albrecht et al., 2006). The bundle characteristics, especially bundle size and fiber number per bundle, are related to muscle growth and meat quality of cattle (Albrecht et al., 2006; Choi et al., 2019). Clear differences in the number and size of muscle bundles and myofiber number per bundle were observed among the cattle breeds that have different muscle characteristics (Albrecht et al., 2006). In our previous study, we reported that a specific line of broiler chickens had a larger bundle CSA of PM muscle with myofiber hypertrophy rather than myofiber number per bundle than a specific line of layers (Kim et al., 2022). As total bundle numbers have not been investigated for both PM and GM muscles of avian species, this is the first study reporting bundle characteristics, including number, size, and myofiber number per bundle, of both PM and GM muscles of broiler and layer chickens. Broilers used in this study had a greater number and CSA of both the muscles, and PM muscle bundles of broilers showed a greater number of myofibers than those of layers (*p* < 0.05). Therefore, PM muscle bundles of broilers are characterized by a greater number and size of muscle bundles with a higher number of myofibers per bundle due to myofiber hyperplasia and hypertrophy. Greater GM muscle mass of broilers is caused by the greater number and size of muscle bundles rather than myofiber number per bundle.

Taken together, broiler and layer chickens show clear differences in histological characteristics of myofibers and muscle bundles of PM and GM muscles. These findings support that fast-growing broilers have greater muscle mass due to both hyperplasia and hypertrophy of myofibers and muscle bundles. Further investigations are needed to identify factors regulating the size and number of muscle bundles and to expand the possible influence of muscle bundles on the understanding of poultry meat quality.

## Data Availability

The original contributions presented in the study are included in the article/Supplementary Material; further inquiries can be directed to the corresponding author.
